# Supplementary material for: Resolving the full spectrum of human genome variation using Linked-Reads
Source: Genome Res. 2019 Apr;29(4):635–45. doi: 10.1101/gr.234443.118 (PMC6442396; doi:10.1101/gr.234443.118)
Supplement: Supplemental Material [file supp_gr.234443.118_Supplemental_longranger-2.2.zip › longranger-2.2.tar.bz2!/lr2.2-public/lib/stan/cmdstan/stan/doc/api/html/neg__binomial__2_8hpp_source.html]

Stan: src/stan/prob/distributions/univariate/discrete/neg\_binomial\_2.hpp Source File


|  |  |
| --- | --- |
|  | Stan  2.5.0  probability, sampling & optimization |


- Main Page
- Related Pages
- Namespaces
- Classes
- Files

- File List
- File Members

All Classes Namespaces Files Functions Variables Typedefs Enumerations Enumerator Friends Macros Pages

- src
- stan
- prob
- distributions
- univariate
- discrete

neg\_binomial\_2.hpp

Go to the documentation of this file.

1 #ifndef STAN\_\_PROB\_\_DISTRIBUTIONS\_\_UNIVARIATE\_\_DISCRETE\_\_NEG\_BINOMIAL\_2\_HPP

2 #define STAN\_\_PROB\_\_DISTRIBUTIONS\_\_UNIVARIATE\_\_DISCRETE\_\_NEG\_BINOMIAL\_2\_HPP

3

4 #include <boost/random/negative\_binomial\_distribution.hpp>

5 #include <boost/random/variate\_generator.hpp>

6

7 #include <boost/math/special\_functions/digamma.hpp>

8 #include <stan/agrad/partials\_vari.hpp>

9 #include <stan/math/error\_handling.hpp>

10 #include <stan/math/constants.hpp>

11 #include <stan/math/functions/multiply\_log.hpp>

12 #include <stan/math/functions/log\_sum\_exp.hpp>

13 #include <stan/math/functions/value\_of.hpp>

14 #include <stan/meta/traits.hpp>

15 #include <stan/prob/traits.hpp>

16 #include <stan/prob/constants.hpp>

17 #include <stan/prob/internal\_math.hpp>

18 #include <stan/prob/distributions/univariate/continuous/gamma.hpp>

19 #include <stan/prob/distributions/univariate/discrete/poisson.hpp>

20 #include <stan/math/functions/binomial\_coefficient\_log.hpp>

21

22 namespace stan {

23

24  namespace prob {

25

26  // NegBinomial(n|mu,phi) [mu >= 0; phi > 0; n >= 0]

27  template <bool propto,

28  typename T\_n,

29  typename T\_location, typename T\_inv\_scale>

30  typename return\_type<T\_location, T\_inv\_scale>::type

31  neg\_binomial\_2\_log(const T\_n& n,

32  const T\_location& mu,

33  const T\_inv\_scale& phi) {

34

35  static const char\* function = "stan::prob::neg\_binomial\_log(%1%)";

36

37  using stan::math::check\_positive\_finite;

38  using stan::math::check\_nonnegative;

39  using stan::math::value\_of;

40  using stan::math::check\_consistent\_sizes;

41  using stan::prob::include\_summand;

42

43  // check if any vectors are zero length

44  if (!(stan::length(n)

45  && stan::length(mu)

46  && stan::length(phi)))

47  return 0.0;

48

49  double logp(0.0);

50  check\_nonnegative(function, n, "Failures variable", &logp);

51  check\_positive\_finite(function, mu, "Location parameter", &logp);

52  check\_positive\_finite(function, phi, "Inverse scale parameter", &logp);

53  check\_consistent\_sizes(function,

54  n,mu,phi,

55  "Failures variable",

56  "Location parameter",

57  "Inverse scale parameter",

58  &logp);

59

60  // check if no variables are involved and prop-to

61  if (!include\_summand<propto,T\_location,T\_inv\_scale>::value)

62  return 0.0;

63

64  using stan::math::multiply\_log;

65  using stan::math::log\_sum\_exp;

66  using stan::math::binomial\_coefficient\_log;

67  using boost::math::digamma;

68  using boost::math::lgamma;

69

70  // set up template expressions wrapping scalars into vector views

71  VectorView<const T\_n> n\_vec(n);

72  VectorView<const T\_location> mu\_vec(mu);

73  VectorView<const T\_inv\_scale> phi\_vec(phi);

74  size\_t size = max\_size(n, mu, phi);

75

76  agrad::OperandsAndPartials<T\_location, T\_inv\_scale>

77  operands\_and\_partials(mu, phi);

78

79  size\_t len\_ep = max\_size(mu, phi);

80  size\_t len\_np = max\_size(n, phi);

81

82  DoubleVectorView<true, is\_vector<T\_location>::value>

83  mu\_\_(length(mu));

84  for (size\_t i = 0, size = length(mu); i < size; ++i)

85  mu\_\_[i] = value\_of(mu\_vec[i]);

86

87  DoubleVectorView<true, is\_vector<T\_inv\_scale>::value>

88  phi\_\_(length(phi));

89  for (size\_t i = 0, size = length(phi); i < size; ++i)

90  phi\_\_[i] = value\_of(phi\_vec[i]);

91

92  DoubleVectorView<true, is\_vector<T\_inv\_scale>::value>

93  log\_phi(length(phi));

94  for (size\_t i = 0, size = length(phi); i < size; ++i)

95  log\_phi[i] = log(phi\_\_[i]);

96

97  DoubleVectorView<true, (is\_vector<T\_location>::value

98  || is\_vector<T\_inv\_scale>::value)>

99  log\_mu\_plus\_phi(len\_ep);

100  for (size\_t i = 0; i < len\_ep; ++i)

101  log\_mu\_plus\_phi[i] = log(mu\_\_[i] + phi\_\_[i]);

102

103  DoubleVectorView<true, (is\_vector<T\_n>::value

104  || is\_vector<T\_inv\_scale>::value)>

105  n\_plus\_phi(len\_np);

106  for (size\_t i = 0; i < len\_np; ++i)

107  n\_plus\_phi[i] = n\_vec[i] + phi\_\_[i];

108

109  for (size\_t i = 0; i < size; i++) {

110  if (include\_summand<propto>::value)

111  logp -= lgamma(n\_vec[i] + 1.0);

112  if (include\_summand<propto,T\_inv\_scale>::value)

113  logp += multiply\_log(phi\_\_[i], phi\_\_[i]) - lgamma(phi\_\_[i]);

114  if (include\_summand<propto,T\_location,T\_inv\_scale>::value)

115  logp -= (n\_plus\_phi[i])\*log\_mu\_plus\_phi[i];

116  if (include\_summand<propto,T\_location>::value)

117  logp += multiply\_log(n\_vec[i], mu\_\_[i]);

118  if (include\_summand<propto,T\_inv\_scale>::value)

119  logp += lgamma(n\_plus\_phi[i]);

120

121  if (!is\_constant\_struct<T\_location>::value)

122  operands\_and\_partials.d\_x1[i]

123  += n\_vec[i]/mu\_\_[i]

124  - (n\_vec[i] + phi\_\_[i])

125  / (mu\_\_[i] + phi\_\_[i]);

126  if (!is\_constant\_struct<T\_inv\_scale>::value)

127  operands\_and\_partials.d\_x2[i]

128  += 1.0 - n\_plus\_phi[i]/(mu\_\_[i] + phi\_\_[i])

129  + log\_phi[i] - log\_mu\_plus\_phi[i] - digamma(phi\_\_[i]) + digamma(n\_plus\_phi[i]);

130  }

131  return operands\_and\_partials.to\_var(logp);

132  }

133

134  template <typename T\_n,

135  typename T\_location, typename T\_inv\_scale>

136  inline

137  typename return\_type<T\_location, T\_inv\_scale>::type

138  neg\_binomial\_2\_log(const T\_n& n,

139  const T\_location& mu,

140  const T\_inv\_scale& phi) {

141  return neg\_binomial\_2\_log<false>(n, mu, phi);

142  }

143

144

145  // NegBinomial(n|eta,phi) [phi > 0; n >= 0]

146  template <bool propto,

147  typename T\_n,

148  typename T\_log\_location, typename T\_inv\_scale>

149  typename return\_type<T\_log\_location, T\_inv\_scale>::type

150  neg\_binomial\_2\_log\_log(const T\_n& n,

151  const T\_log\_location& eta,

152  const T\_inv\_scale& phi) {

153

154  static const char\* function = "stan::prob::neg\_binomial\_log(%1%)";

155

156  using stan::math::check\_finite;

157  using stan::math::check\_nonnegative;

158  using stan::math::check\_positive\_finite;

159  using stan::math::value\_of;

160  using stan::math::check\_consistent\_sizes;

161  using stan::prob::include\_summand;

162

163  // check if any vectors are zero length

164  if (!(stan::length(n)

165  && stan::length(eta)

166  && stan::length(phi)))

167  return 0.0;

168

169  double logp(0.0);

170  check\_nonnegative(function, n, "Failures variable", &logp);

171  check\_finite(function, eta, "Log location parameter", &logp);

172  check\_positive\_finite(function, phi, "Inverse scale parameter", &logp);

173  check\_consistent\_sizes(function,

174  n,eta,phi,

175  "Failures variable",

176  "Log location parameter",

177  "Inverse scale parameter",

178  &logp);

179

180  // check if no variables are involved and prop-to

181  if (!include\_summand<propto,T\_log\_location,T\_inv\_scale>::value)

182  return 0.0;

183

184  using stan::math::multiply\_log;

185  using stan::math::log\_sum\_exp;

186  using stan::math::binomial\_coefficient\_log;

187  using boost::math::digamma;

188  using boost::math::lgamma;

189

190  // set up template expressions wrapping scalars into vector views

191  VectorView<const T\_n> n\_vec(n);

192  VectorView<const T\_log\_location> eta\_vec(eta);

193  VectorView<const T\_inv\_scale> phi\_vec(phi);

194  size\_t size = max\_size(n, eta, phi);

195

196  agrad::OperandsAndPartials<T\_log\_location,T\_inv\_scale>

197  operands\_and\_partials(eta,phi);

198

199  size\_t len\_ep = max\_size(eta, phi);

200  size\_t len\_np = max\_size(n, phi);

201

202  DoubleVectorView<true, is\_vector<T\_log\_location>::value>

203  eta\_\_(length(eta));

204  for (size\_t i = 0, size = length(eta); i < size; ++i)

205  eta\_\_[i] = value\_of(eta\_vec[i]);

206

207  DoubleVectorView<true, is\_vector<T\_inv\_scale>::value>

208  phi\_\_(length(phi));

209  for (size\_t i = 0, size = length(phi); i < size; ++i)

210  phi\_\_[i] = value\_of(phi\_vec[i]);

211

212

213  DoubleVectorView<true, is\_vector<T\_inv\_scale>::value>

214  log\_phi(length(phi));

215  for (size\_t i = 0, size = length(phi); i < size; ++i)

216  log\_phi[i] = log(phi\_\_[i]);

217

218  DoubleVectorView<true, (is\_vector<T\_log\_location>::value

219  || is\_vector<T\_inv\_scale>::value)>

220  logsumexp\_eta\_logphi(len\_ep);

221  for (size\_t i = 0; i < len\_ep; ++i)

222  logsumexp\_eta\_logphi[i] = log\_sum\_exp(eta\_\_[i], log\_phi[i]);

223

224  DoubleVectorView<true, (is\_vector<T\_n>::value

225  || is\_vector<T\_inv\_scale>::value)>

226  n\_plus\_phi(len\_np);

227  for (size\_t i = 0; i < len\_np; ++i)

228  n\_plus\_phi[i] = n\_vec[i] + phi\_\_[i];

229

230  for (size\_t i = 0; i < size; i++) {

231  if (include\_summand<propto>::value)

232  logp -= lgamma(n\_vec[i] + 1.0);

233  if (include\_summand<propto,T\_inv\_scale>::value)

234  logp += multiply\_log(phi\_\_[i], phi\_\_[i]) - lgamma(phi\_\_[i]);

235  if (include\_summand<propto,T\_log\_location,T\_inv\_scale>::value)

236  logp -= (n\_plus\_phi[i])\*logsumexp\_eta\_logphi[i];

237  if (include\_summand<propto,T\_log\_location>::value)

238  logp += n\_vec[i]\*eta\_\_[i];

239  if (include\_summand<propto,T\_inv\_scale>::value)

240  logp += lgamma(n\_plus\_phi[i]);

241

242  if (!is\_constant\_struct<T\_log\_location>::value)

243  operands\_and\_partials.d\_x1[i]

244  += n\_vec[i] - n\_plus\_phi[i]

245  / (phi\_\_[i]/exp(eta\_\_[i]) + 1.0);

246  if (!is\_constant\_struct<T\_inv\_scale>::value)

247  operands\_and\_partials.d\_x2[i]

248  += 1.0 - n\_plus\_phi[i]/(exp(eta\_\_[i]) + phi\_\_[i])

249  + log\_phi[i] - logsumexp\_eta\_logphi[i] - digamma(phi\_\_[i]) + digamma(n\_plus\_phi[i]);

250  }

251  return operands\_and\_partials.to\_var(logp);

252  }

253

254  template <typename T\_n,

255  typename T\_log\_location, typename T\_inv\_scale>

256  inline

257  typename return\_type<T\_log\_location, T\_inv\_scale>::type

258  neg\_binomial\_2\_log\_log(const T\_n& n,

259  const T\_log\_location& eta,

260  const T\_inv\_scale& phi) {

261  return neg\_binomial\_2\_log\_log<false>(n,eta,phi);

262  }

263

264  template <class RNG>

265  inline int

266  neg\_binomial\_2\_rng(const double mu,

267  const double phi,

268  RNG& rng) {

269  using boost::variate\_generator;

270  using boost::random::negative\_binomial\_distribution;

271

272  static const char\* function = "stan::prob::neg\_binomial\_2\_rng(%1%)";

273

274  using stan::math::check\_positive\_finite;

275

276  check\_positive\_finite(function, mu, "Location parameter", (double\*)0);

277  check\_positive\_finite(function, phi, "Inverse scale parameter",

278  (double\*)0);

279

280  return stan::prob::poisson\_rng(stan::prob::gamma\_rng(phi,phi/mu,

281  rng),rng);

282  }

283

284  template <class RNG>

285  inline int

286  neg\_binomial\_2\_log\_rng(const double eta,

287  const double phi,

288  RNG& rng) {

289  using boost::variate\_generator;

290  using boost::random::negative\_binomial\_distribution;

291

292  static const char\* function = "stan::prob::neg\_binomial\_2\_log\_rng(%1%)";

293

294  using stan::math::check\_finite;

295  using stan::math::check\_positive\_finite;

296

297  check\_finite(function, eta, "Log-location parameter", (double\*)0);

298  check\_positive\_finite(function, phi, "Inverse scale parameter",

299  (double\*)0);

300

301  return stan::prob::poisson\_rng(stan::prob::gamma\_rng(phi,phi/std::exp(eta),

302  rng),rng);

303  }

304  }

305 }

306 #endif

log\_sum\_exp.hpp

multiply\_log.hpp

stan::agrad::log\_sum\_exp

fvar< T > log\_sum\_exp(const fvar< T > &x1, const fvar< T > &x2)

**Definition:** log\_sum\_exp.hpp:15

traits.hpp

stan::math::log\_sum\_exp

boost::math::tools::promote\_args< T1, T2 >::type log\_sum\_exp(const T2 &a, const T1 &b)

Calculates the log sum of exponetials without overflow.

**Definition:** log\_sum\_exp.hpp:49

stan::agrad::OperandsAndPartials::to\_var

T\_return\_type to\_var(double logp)

**Definition:** partials\_vari.hpp:138

stan::math::check\_positive\_finite

bool check\_positive\_finite(const char \*function, const T\_y &y, const char \*name, T\_result \*result)

**Definition:** check\_positive\_finite.hpp:12

stan::prob::neg\_binomial\_2\_log\_rng

int neg\_binomial\_2\_log\_rng(const double eta, const double phi, RNG &rng)

**Definition:** neg\_binomial\_2.hpp:286

error\_handling.hpp

stan::math::multiply\_log

boost::math::tools::promote\_args< T\_a, T\_b >::type multiply\_log(const T\_a a, const T\_b b)

Calculated the value of the first argument times log of the second argument while behaving properly w...

**Definition:** multiply\_log.hpp:51

poisson.hpp

stan::length

size\_t length(const T &)

**Definition:** traits.hpp:159

stan::math::binomial\_coefficient\_log

boost::math::tools::promote\_args< T\_N, T\_n >::type binomial\_coefficient\_log(const T\_N N, const T\_n n)

Return the log of the binomial coefficient for the specified arguments.

**Definition:** binomial\_coefficient\_log.hpp:63

stan::DoubleVectorView

DoubleVectorView allocates double values to be used as intermediate values.

**Definition:** traits.hpp:358

stan::math::check\_finite

bool check\_finite(const char \*function, const T\_y &y, const char \*name, T\_result \*result)

Checks if the variable y is finite.

**Definition:** check\_finite.hpp:52

partials\_vari.hpp

stan::agrad::value\_of

T value\_of(const fvar< T > &v)

Return the value of the specified variable.

**Definition:** value\_of.hpp:16

stan::prob::neg\_binomial\_2\_rng

int neg\_binomial\_2\_rng(const double mu, const double phi, RNG &rng)

**Definition:** neg\_binomial\_2.hpp:266

constants.hpp

stan::is\_vector

**Definition:** traits.hpp:83

stan::agrad::lgamma

fvar< T > lgamma(const fvar< T > &x)

**Definition:** lgamma.hpp:15

stan::agrad::OperandsAndPartials

A variable implementation that stores operands and derivatives with respect to the variable...

**Definition:** partials\_vari.hpp:76

traits.hpp

stan::return\_type::type

boost::math::tools::promote\_args< typename scalar\_type< T1 >::type, typename scalar\_type< T2 >::type, typename scalar\_type< T3 >::type, typename scalar\_type< T4 >::type, typename scalar\_type< T5 >::type, typename scalar\_type< T6 >::type >::type type

**Definition:** traits.hpp:406

stan::is\_constant\_struct

Metaprogram to determine if a type has a base scalar type that can be assigned to type double...

**Definition:** traits.hpp:57

stan::math::value\_of

double value\_of(const T x)

Return the value of the specified scalar argument converted to a double value.

**Definition:** value\_of.hpp:24

stan::prob::neg\_binomial\_2\_log\_log

return\_type< T\_log\_location, T\_inv\_scale >::type neg\_binomial\_2\_log\_log(const T\_n &n, const T\_log\_location &eta, const T\_inv\_scale &phi)

**Definition:** neg\_binomial\_2.hpp:150

stan::prob::include\_summand

Template metaprogram to calculate whether a summand needs to be included in a proportional (log) prob...

**Definition:** traits.hpp:35

stan::prob::neg\_binomial\_2\_log

return\_type< T\_location, T\_inv\_scale >::type neg\_binomial\_2\_log(const T\_n &n, const T\_location &mu, const T\_inv\_scale &phi)

**Definition:** neg\_binomial\_2.hpp:31

stan::prob::poisson\_rng

int poisson\_rng(const double lambda, RNG &rng)

**Definition:** poisson.hpp:384

stan::agrad::OperandsAndPartials::d\_x2

VectorView< double \*, is\_vector< T2 >::value, is\_constant\_struct< T2 >::value > d\_x2

**Definition:** partials\_vari.hpp:83

stan::math::check\_nonnegative

bool check\_nonnegative(const char \*function, const T\_y &y, const char \*name, T\_result \*result)

**Definition:** check\_nonnegative.hpp:52

stan::math::check\_consistent\_sizes

bool check\_consistent\_sizes(const char \*function, const T1 &x1, const T2 &x2, const char \*name1, const char \*name2, T\_result \*result)

**Definition:** check\_consistent\_sizes.hpp:13

stan::max\_size

size\_t max\_size(const T1 &x1, const T2 &x2)

**Definition:** traits.hpp:191

internal\_math.hpp

stan::agrad::multiply\_log

fvar< T > multiply\_log(const fvar< T > &x1, const fvar< T > &x2)

**Definition:** multiply\_log.hpp:15

stan::math::size

int size(const std::vector< T > &x)

**Definition:** size.hpp:11

stan::agrad::digamma

fvar< T > digamma(const fvar< T > &x)

**Definition:** digamma.hpp:16

value\_of.hpp

stan::agrad::OperandsAndPartials::d\_x1

VectorView< double \*, is\_vector< T1 >::value, is\_constant\_struct< T1 >::value > d\_x1

**Definition:** partials\_vari.hpp:82

binomial\_coefficient\_log.hpp

constants.hpp

stan::agrad::log

fvar< T > log(const fvar< T > &x)

**Definition:** log.hpp:15

stan::VectorView

VectorView is a template metaprogram that takes its argument and allows it to be used like a vector...

**Definition:** traits.hpp:275

stan::agrad::exp

fvar< T > exp(const fvar< T > &x)

**Definition:** exp.hpp:16

stan::prob::gamma\_rng

double gamma\_rng(const double alpha, const double beta, RNG &rng)

**Definition:** gamma.hpp:491

gamma.hpp


---

[ Stan Home Page ]

*© 2011–2014,
Stan Development Team.*
